# The total prevalence of diagnosed diabetes and the quality of diabetes care for the adult population in Salten, Norway

**DOI:** 10.1177/1403494820951004

**Published:** 2020-08-27

**Authors:** Kristina B. Slåtsve, Tor Claudi, Knut Tore Lappegård, Anne K. Jenum, Marthe Larsen, John G. Cooper, Sverre Sandberg, Tore Julsrud Berg

**Affiliations:** 1 Department of Medicine, Nordland Hospital, Norway; 2 Department of Clinical Medicine, The Arctic University of Norway, Norway; 3 General Practice Research Unit (AFE), University of Oslo, Norway; 4 Clinical Research Department, University Hospital of North Norway, Norway; 5 Department of Medicine, Stavanger University Hospital, Norway; 6 Norwegian Quality Improvement of Laboratory Examinations, Haraldsplass Deaconess Hospital, Norway; 7 Department of Public Health and Primary Health Care, University of Bergen, Norway; 8 Department of Clinical Biochemistry, Haukeland University Hospital, Norway; 9 Institute of Clinical Medicine, University of Oslo, Norway; 10 Department of Endocrinology, Morbid Obesity and Preventive Medicine, Oslo University Hospital, Norway

**Keywords:** Diabetes, type 1 diabetes, type 2 diabetes, prevalence, vascular complication, primary healthcare

## Abstract

*Objective:* To assess the total prevalence of types 1 and 2 diabetes and to describe and compare cardiovascular risk factors, vascular complications and the quality of diabetes care in adults with types 1 and 2 diabetes in Salten, Norway. *Research design and methods:* Cross-sectional study including all patients with diagnosed diabetes in primary and specialist care in Salten, 2014 (population 80,338). Differences in cardiovascular risk factors, prevalence of vascular complications and attained treatment targets between diabetes types were assessed using regression analyses. *Results:* We identified 3091 cases of diabetes, giving a total prevalence in all age groups of 3.8%, 3.4% and 0.45% for types 2 and 1 diabetes, respectively. In the age group 30–89 years the prevalence of type 2 diabetes was 5.3%. Among 3027 adults aged 18 years and older with diabetes, 2713 (89.6%) had type 2 and 304 (10.0%) type 1 diabetes. The treatment target for haemoglobin A1c (⩽7.0%/53 mmol/mol) was reached in 61.1% and 22.5% of types 2 and 1 diabetes patients, respectively. After adjusting for age, sex and diabetes duration we found differences between patients with types 2 and 1 diabetes in mean haemoglobin A1c (7.1% vs. 7.5%, *P*<0.001), blood pressure (136/78 mmHg vs. 131/74 mmHg, *P*<0.001) and prevalence of coronary heart disease (23.1% vs. 15.8%, *P*<0.001). *Conclusions:* The prevalence of diagnosed type 2 diabetes was slightly lower than anticipated. Glycaemic control was not satisfactory in the majority of patients with type 1 diabetes. Coronary heart disease was more prevalent in patients with type 2 diabetes.

## Significance of this study

### What is already known about this subject?

Studies reporting the prevalence of diabetes in Norway and worldwide have mostly been based on self-reported data, diagnoses in electronic medical records, registry data, or the use of blood glucose-lowering drugs.

### What are the new findings?

Based on validated data collected from all physicians treating individuals with diabetes in a geographically defined area, the total prevalence of diagnosed type 2 and type 1 diabetes in Salten was 3.4% and 0.45%, respectively. More type 2 diabetes patients than type 1 diabetes patients reached the haemoglobin A1c (HbA1c) treatment target. The patterns of cardiovascular risk factors (HbA1c and blood pressure) differed significantly between type 2 and type 1 diabetes patients. Patients with type 2 diabetes had lower mean HbA1c, whereas patients with type 1 diabetes had lower mean blood pressure. The adjusted prevalence of coronary heart disease (CHD) was 23.1% and 15.8% in type 2 and type 1 diabetes patients, respectively.

## How might these results change the focus of research or clinical practice?

We found a slightly lower prevalence of diabetes than anticipated. Furthermore, we identified quality gaps in the treatment that differed by type of diabetes. This knowledge can be used in quality improvement strategies.

## Introduction

Type 1 and type 2 diabetes are complex metabolic diseases that differ in pathophysiology and treatment. The global prevalence of diabetes in adults (age 18–99 years) in 2017 was estimated to be 8.4% and a worrisome increase is predicted worldwide in the coming years [1]. Pooled data from population-based studies found a global age-standardised diabetes prevalence of 9.0% in men and 7.9% in women in 2014 [2]. In Norway the prevalence of type 2 diabetes was reported to be 6.1% (age 30–89 years) in 2014 [3].

Compared to people without diabetes, patients with type 2 diabetes have a 15% increased risk of all-cause mortality, and the mortality is higher in younger age groups [4]. Inadequate glycaemic control, hypertension, elevated levels of low-density lipoprotein (LDL) cholesterol and smoking are established risk factors for cardiovascular disease (CVD) shown to be reduced by improved management of diabetes [5,6]. Repeated Norwegian cross-sectional surveys have shown improvements in the achievement of diabetes treatment targets over time [7]. Although the treatment targets are identical in type 1 and type 2 diabetes, identifying subgroups in need of closer follow-up and overcoming the barriers achieving treatment targets will be more important in the coming years.

There is a lack of real-world data describing the total population with diagnosed diabetes within a geographical area with validated clinical data. We hypothesise that the prevalence of diagnosed diabetes differs from studies based on self-reported data, administrative registries without validated diagnoses, or health surveys with a risk of selection bias. Our first objective was therefore to describe the prevalence of diagnosed type 1 and type 2 diabetes in all age groups in the geographical area of Salten, Norway. Furthermore, we aimed to identify gaps in the quality of care for type 1 and type 2 diabetes patients in this population by comparing cardiovascular risk factors, vascular complications and attained treatment targets according to national guidelines.

## Research design and methods

The present cross-sectional study is part of the ROSA 4 (Rogaland-Oslo-Salten-Akershus-Hordaland) study, assessing the quality of diabetes care within an integrated healthcare system in 2014 [7]. The study was approved by the Regional Ethical Committee West (REK 2014/1374, REK Vest), with permission to collect data from general practice without written consent. Data from the outpatient clinic included patients consenting to send their data to the Norwegian Diabetes Registry for Adults.

The public healthcare system in Norway is financed through government funding. Every citizen has the right to be registered with a general practitioner (GP). Residents aged 16 years and older must pay an annual deductible, in 2014 approximately €233 for doctors’ visits and drug prescriptions before getting free essential drugs and appointments in primary and specialist care. In-hospital treatments are free.

### Setting

The Salten region in Northern Norway has a total population of 80,338 as of 31 December 2014, covers approximately 10,000 km^2^, nine municipalities and one town (approximately 50,000 inhabitants). A diabetes action plan was launched in 2009 facilitating a close collaboration between GPs in the area and the diabetes outpatient clinic at the only hospital that serves all diabetes patients in need of specialist care. There are no private diabetologists in the region. In 2014 the prevalence of immigrants born outside Norway was lower in Salten than in Norway as a whole (7.1% vs. 12.4%). The proportions of immigrants from Africa and Asia were 1.3% and 1.5% in Salten (compared to 1.7% and 3.5%, in Norway), respectively.

### Data collection

To be able to include all patients with diabetes living in Salten, we used four independent data sources. First, data collected from primary care included all patients with known diabetes visiting a GP from 1 January 2012 to 31 December 2014. All GPs (*n*=82) were invited to take part in the study and all accepted. The data collection was facilitated using a software program from the Norwegian Diabetes Registry for Adults, which identified all adults (⩾18 years) with a diagnosis of diabetes (T89 and T90 in the International Classification of Primary Care (ICPC)) in the defined time period. Predefined variables were extracted from the electronic medical records for each patient. A research nurse scrutinised all primary care electronic medical records including mandatory copies of patient reports from all types of specialist care visits, to verify electronically captured data and collect missing data not suitable for electronic capture. The data collection was performed from April to December 2015. Second, relevant data from all adult patients with diabetes visiting the hospital diabetes outpatient clinic from 31 October 2013 to 31 December 2014 were collected. Third, information on the number of patients with diabetes in the paediatric population was obtained from the paediatric clinic at the same hospital. Fourth, each municipality included in the study was contacted by phone to provide information about the number of people permanently living in nursing homes with no follow-up by a GP.

### Variables

Diabetes was categorised as type 1 diabetes including latent autoimmune diabetes of adults (LADA), type 2 diabetes and by other types (including maturity-onset diabetes of the young (MODY) or pancreatitis). The diagnosis of diabetes type was based on the doctor’s clinical diagnosis supported by measurements of beta cell antibodies and C-peptide when necessary. Information on patient characteristics, processes of care, intermediate outcomes, complications, medication and information on GPs and GP practices was registered. For the majority of patient variables, we included the last registered value in the period 1 October 2013 to 31 December 2014 (Supplemental Table I). Eye examination, creatinine/estimated glomerular filtration rate (eGFR) and lipids were registered for the period 1 January 2012 to 31 December 2014 and smoking habits 2010–2014. Data from the most recent visit were used in the analyses. If data in patients visiting both primary and specialist care clinic differed, the most adverse or recent outcome/complication was used.

Type 1 and type 2 diabetes treatment targets were identical and based on the Norwegian national treatment guidelines from 2009: HbA1c 7.0% or less (53 mmol/mol); intervention threshold for blood pressure greater than 140/85 mmHg with treatment target of 135/80 mmHg or less; total cholesterol 4.5 mmol/L or less and LDL-cholesterol 3.5 mmol/L or less with treatment target for LDL-cholesterol 1.8 mmol/L or less and 2.5 mmol/L or less for individuals with and without known CHD, respectively [8].

### Statistical analyses

To estimate the crude prevalence of diabetes, we used the total number of diabetes cases identified, including number of cases from the paediatric clinic as the nominator.

The denominator was the total number of individuals alive and residing in each of the nine municipalities in Salten by 31 December 2014 according to Statistics Norway. The prevalence estimates were stratified by diabetes type, 10-year age groups and sex. We also estimated the total prevalence using the proportion of immigrants in Norway and by including the estimated number of people with diabetes permanently living in nursing homes.

Descriptive statistics are presented as percentages, means with standard deviations (SDs) or medians with interquartile range (IQR). Bivariate parametric and non-parametric tests were used as appropriate.

Both univariable and multivariable linear and logistic regression models were used to compare variables of interest between diabetes types. In the multivariable models, we adjusted for age, sex and diabetes duration due to possible confounding between diabetes type and the outcomes of interest. We present average adjusted predictions (AAPs) and average marginal effects (AMEs) with 95% confidence intervals (CIs) and *P* values from univariable and multivariable regression. Crude attained treatment targets are presented in figures and AAPs for attained treatment targets are presented in the text. The significance level was set at 0.05 for all analyses. All statistical analyses were performed using STATA/SE 14 (StataCorp LP, College Station, Texas, USA).

After excluding duplicates, patients with gestational diabetes, patients who were not registered with an address or not residing in Salten, and those registered as dead (*n*=4), we studied 3035 adults with diabetes. Furthermore, 56 children (<18 years), all with type 1 diabetes, were included in the sample of 3091 persons used to calculate the total prevalence (Supplemental Figure 1). In 2014, the total number of people permanently living in nursing homes was 570, and we estimated the number of people with diabetes in this population to be 90–95 [9].

The clinical dataset of adults used in further analyses included 3027 patients obtained from 82 GPs in 26 practices (100% of the invited) and all consenting patients (*n*=604, 98.7%) visiting the diabetes outpatient clinic (Supplemental Figure 2). Age-adjusted prevalence was calculated by adding the number of children with diabetes to this dataset, giving a sample of 3083 patients.

## Results

### Prevalence of diabetes

**Figure 1.**
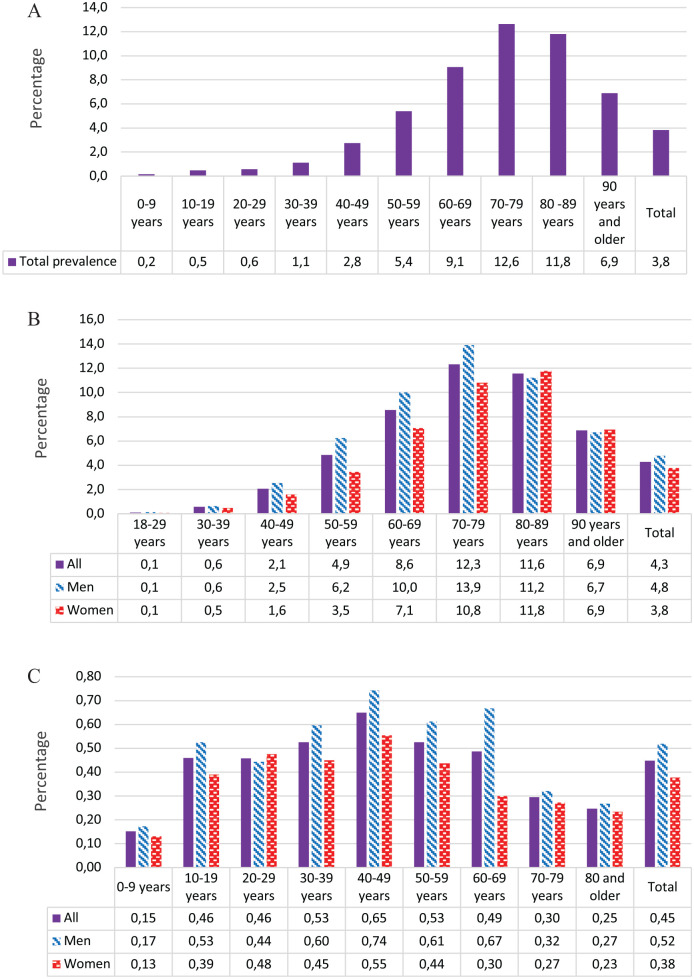
Total prevalence of diabetes and by diabetes type, %. (a) Total prevalence, all diabetes types, %. (b) Type 2 diabetes prevalence, %. (c) Type 1 diabetes prevalence, %.

The total prevalence of diagnosed diabetes was 3.8% and increased with age up to 80 years (Figure 1). In adults aged 20 years and older the prevalence was 4.9%. The overall prevalence of type 2 diabetes (all age groups) was 3.4%; 4.4% in those aged 20 years and older and 5.3% in the age group 30–89 years. Type 2 diabetes was more prevalent in men than in women in all age groups. The prevalence of type 1 diabetes (all age groups) and in the age group 20 years and older was 0.45% and 0.49%, respectively.

When we extrapolated the proportion of immigrants from Asia and Africa in Norway to diabetes prevalence in Salten, with a prevalence of diabetes in this group set to 15%, the prevalence of diabetes in the 30–89 years age group increased marginally from 5.3% to 5.4% [10]. Age standardisation using age distribution from the Norwegian population by 31 December 2014 did not change the prevalence estimates. Including the estimated number of people with type 2 diabetes permanently living in nursing homes changed the total prevalence estimate (all age groups) from 3.8% to 3.9–4.0%.

### Characteristics of adults with diabetes

Type 2 and type 1 diabetes accounted for 89.6% and 10.0%, respectively. Ten patients (0.3%) had other types of diabetes. The sample included 2713 patients with type 2 diabetes with a mean age of 67 years, median diabetes duration of 7 years (Table I) and a mean body mass index (BMI) of 30.5 kg/m^2^. The majority (56.4%) of type 2 diabetes patients were men and they were younger than women, also at the time of diagnosis. Among the 304 patients with type 1 diabetes, the mean age was 47 years and median diabetes duration of 19 years.

**Table I.** Characteristics and vascular complications of all adults with diabetes in Salten, Norway.

	Type 2 diabetes, *n*=2713				Type 1 diabetes, *n*=304			
	Valid data, *n* (%)	All	Men	Women	Valid data, *n* (%)	All	Men	Women
**Patient characteristics**								
Sex, *n* (%)	2713 (100)		1530 (56.4)	1183 (43.6)	304 (100)		177 (58.2)	127 (41.8)
Age (years), mean (SD)	2713 (100)	65.6 (12.7)	64.0 (12.1)	67.7 (13.2)	304 (100)	46.7 (15.7)	47.2 (15.3)	45.9 (16.4)
Age at diagnosis (years), mean (SD)	2522 (93.0)	56.9 (12.5)	55.6 (12.0)	58.7 (12.9)	300 (98.7)	25.9 (17.2)	25.8 (17.0)	25.9 (17.7)
BMI (kg/m^2^), mean (SD)	1532 (56.4)	30.5 (6.0)	30.4 (5.9)	30.6 (6.2)	282 (92.8)	26.5 (4.5)	26.5 (4.1)	26.6 (5.1)
Diabetes duration (years), median (IQR)	2522 (93.0)	7 (3–12)	7 (3–12)	7 (4–13)	300 (98.7)	19 (11–30)	20 (11–31)	18 (9–29)
Attending hospital outpatient clinic, *n* (%)	2713 (100)	348 (12.8)	225 (14.7)	123 (10.4)	304 (100)	107 (84.3)	142 (80.2)	107 (84.3)
**Complications**								
Macrovascular complications, *n* (%)	2680 (98.8)	782 (29.2)	530 (35.0)	252 (21.6)	284 (93.4)	37 (13.0)	24 (14.5)	13 (11.0)
Coronary heart disease, *n* (%)	2684 (98.9)	646 (24.1)	443 (29.2)	203 (17.4)	284 (93.4)	29 (10.2)	19 (11.5)	10 (8.5)
Stroke, *n* (%)	2690 (99.2)	206 (7.7)	133 (8.8)	73 (6.2)	284 (93.4)	12 (4.2)	8 (4.8)	4 (3.4)
PTA/art. surgery, *n* (%)	2680 (98.8)	67 (2.5)	47 (3.1)	20 (1.7)	284 (93.4)	9 (3.2)	6 (3.6)	3 (2.5)
History of foot ulcer, *n* (%)	2686 (99.0)	62 (2.3)	39 (2.6)	23 (2.0)	283 (93.1)	18 (6.4)	12 (7.2)	6 (5.1)
Lower limb amputations, *n* (%)	2690 (99.2)	27 (1.0)	20 (1.3)	7 (0.6)	284 (93.4)	6 (2.1)	5 (3.0)	1 (0.9)
Retinopathy, all *n* (%)	2088 (77.0)	243 (11.6)	169 (14.1)	74 (8.3)	266 (87.5)	139 (52.3)	90 (59.2)	49 (43.0)
Untreated	–	194 (9.3)	133 (11.1)	61 (6.9)	–	93 (35.0)	61 (40.1)	32 (28.1)
Treated	–	49 (2.4)	36 (3.0)	13 (1.5)	–	46 (17.3)	29 (19.1)	17 (14.9)
Nephropathy, (eGFR, ml/min), *n* (%)	2594 (95.6)	–	–	–	294 (96.7)	–	–	–
≥60		2178 (84.0)	1264 (86.9)	914 (80.3)	–	283 (96.3)	165 (98.2)	118 (93.7)
30–59	–	365 (14.1)	1665 (11.3)	200 (17.6)	–	9 (3.1)	2 (1.2)	7 (5.6)
<30	–	51 (2.0)	26 (1.8)	25 (2.2)	–	2 (0.7)	1 (0.6)	1 (0.8)

Data are presented as means with standard deviation (SD), median with interquartile range (IQR) or percentage.

BMI: body mass index; eGFR: estimated glomerular filtration rate; Macrovascular complications: coronary heart disease (angina/myocardial infarction/percutaneous coronary intervention/bypass), stroke, percutaneous transluminal angioplasty or arterial surgery; PTA/art. surgery: percutaneous transluminal angioplasty or arterial surgery.

In the total dataset, 2423 patients (80.1%) had their follow-up in primary care only, 109 (3.6%) in hospital outpatient clinic only and 495 (16.4%) had shared care (Supplemental Figure 2).

### Prevalence of vascular complications

The crude prevalence of any macrovascular complication and CHD was higher in type 2 diabetes than in type 1 diabetes patients, while the prevalence of diagnosed retinopathy was substantially higher in patients with type 1 diabetes (Table II). After adjustments for age, sex and diabetes duration, CHD remained significantly more prevalent in type 2 than in type 1 diabetes patients (23.1% vs. 15.8%, *P*=0.019), whereas retinopathy differences became borderline significant. Moreover, 0.7% of type 2 and 0.5% of type 1 diabetes patients were in dialysis, and 0.3% of type 2 and 0.9% of type 1 diabetes patients had undergone kidney transplantation. Information was registered in 48.9% type 2 diabetes and 70.4% type 1 diabetes patients.

**Table II.** Observed and adjusted vascular complications of all adults with diabetes in Salten, Norway.

**Complications**	Type 2, *n*=2713		Type 1, *n*=304		Observed difference	Adjusted difference^b^	*P* value margins, observed	*P* value margins, adjusted
	Observed	Adjusted^a^	Observed	Adjusted^a^				
Macro-vascular complications, % (95% CI)	29.2 (27.5 to 31.0)	27.9 (26.3 to 29.5)	13.0 (9.1 to 16.9)	22.4 (15.7 to 29.1)	16.2 (11.9 to 20.4)	5.5 (−1.5 to 12.5)	<0.001	0.123
Coronary heart disease, % (95% CI)	24.1 (22.5 to 25.7)	23.1 (21.6 to 24.7)	10.2 (6.7 to 13.7)	15.8 (10.0 to 1.6)	13.9 (10.1 to 17.7)	7.3 (1.2 to 13.5)	<0.001	0.019
Stroke, % (95% CI)	7.7 (6.7 to 8.7)	7.3 (6.4 to 8.3)	4.2 (1.9 to 6.6)	9.9 (4.3 to 15.5)	3.4 (0.9 to 6.0)	−2.5 (−8.3 to 3.2)	0.008	0.384
PTA/art. surgery, % (95% CI)	2.5 (1.9 to 3.1)	2.7 (2.0 to 3.3)	3.2 (1.1 to 5.2)	3.0 (0.4 to 5.5)	−0.7 (−2.8 to 1.5)	−0.3 (−3.0 to 2.4)	0.536	0.841
Foot ulcer, % (95% CI)	2.3 (1.7 to 2.9)	2.8 (2.0 to 3.7)	6.4 (3.5 to 9.2)	2.5 (0.8 to 4.3)	−4.1 (−7.0 to −1.2)	0.3 (−1.8 to 2.5)	0.006	0.774
Lower limb amputations, % (95% CI)	1.0 (0.6 to 1.4)	1.1 (0.6 to 1.7)	2.1 (0.4 to 3.8)	1.0 (−0.1 to 2.2)	−1.1 (−2.8 to 0.6)	0.1 (−1.3 to 1.6)	0.205	0.843
Retinopathy, % (95% CI)	11.6 (10.3 to 13.0)	15.5 (13.9 to 17.0)	52.3 (46.3 to 58.3)	21.0 (16.0 to 25.9)	−40.6 (−46.8 to −34.5)	−5.5 (−11.1 to 0.0)	<0.001	0.052
Nephropathy, (eGFR, ml/min), mean (95% CI)	83 (82 to 84)	85 (84 to 86)	103 (100 to 105)	87 (85 to 89)	−20 (−22 to −17)	−2 (−4 to 0)	<0.001	0.116

a Average predictions adjusted for age, sex and diabetes duration.

b Average marginal effects.

CI: confidence interval; eGFR; estimated glomerular filtration rate; Macrovascular complications: coronary heart disease (angina/myocardial infarction/percutaneous coronary intervention/bypass), stroke, percutaneous transluminal angioplasty or arterial surgery; PTA/art. surgery: percutaneous transluminal angioplasty or arterial surgery.

### Cardiovascular risk factors and prescriptions of blood glucose-lowering medications

After adjusting for age, sex and diabetes duration, we found differences in mean HbA1c (7.1% vs. 7.5%, *P*<0.001) and blood pressure (136/78 mmHg vs. 131/74 mmHg, *P*<0.001) but not in LDL-cholesterol between patients with type 2 and type 1 diabetes (Table III). The proportion of current smokers was 18.6% in both type 2 and type 1 diabetes patients.

**Table III.** Observed and adjusted cardiovascular risk factors and prescribed medication in all adults with diabetes in Salten, Norway.

	Type 2 (*N*=2713)			Type 1 (*N*=304)			Adjusted difference^c^	*P* value for adjusted
	Valid numbers (%)	Observed	Adjusted^b^	Valid numbers (%)	Observed	Adjusted^b^		
HbA1c, %, mean (95% CI)	2435 (89.8)	7.0 (7.0 to 7.1)	7.1 (7.0 to 7.2)	289 (95.1)	8.1 (8.0 to 8.3)	7.5 (7.4 to 7.7)	−0.4 (−0.6 to −0.3)	<0.001
HbA1c, mmol/mol, mean (95% CI)	2435 (89.8)	53 (53 to 54)	54 (54 to 55)	289 (95.1)	66 (64 to 67)	59 (57 to 61)	−4.8 (−6.8 to −2.7)	<0.001
Systolic blood pressure, mmHg, mean (95% CI)	2277 (83.9)	137 (137 to 138)	136 (136 to 137)	288 (94.7)	127 (126 to 129)	131 (129 to 133)	5.4 (2.9 to 8.0)	<0.001
Diastolic blood pressure, mmHg, mean (95% CI)	2277 (83.9)	78 (78 to 78)	78 (78 to 78)	288 (94.7)	74 (73 to 75)	74 (72 to 75)	4.5 (3.0 to 5.9)	<0.001
LDL-cholesterol, mmol/l, mean (95% CI)	2408 (88.8)	2.8 (2.7 to 2.8)	2.8 (2.7 to 2.8)	288 (94.7)	2.7 (2.6 to 2.8)	2.8 (2.6 to 2.9)	−0.0 (−0.1 to 0.1)	0.915
With CHD, mmol/l	587 (21.6^1^) (90.9^2^)	2.5 (2.4 to 2.5)	2.5 (2.4 to 2.5)	29 (9.5^1^) (100^2^)	2.1 (1.9 to 2.4)	2.1 (1.7 to 2.4)	0.4 (0.0 to 0.8)	0.051
No CHD, mmol/l	1795 (66.2^1^) (88.1^2^)	2.9 (2.8 to 2.9)	2.8 (2.8 to 2.9)	240 (78.9^1^) (94.1^2^)	2.8 (2.7 to 2.9)	2.9 (2.8 to 3.1)	−0.1 (−0.2 to 0.1)	0.242
Prescribed lipid-lowering agents, mmol/l	1345 (49.6^1^) (96.1^3^)	2.5 (2.5 to 2.6)	2.5 (2.4 to 2.5)	98 (32.2^1^) (100^3^)	2.5 (2.3 to 2.7)	2.7 (2.5 to 2.8)	−0.2 (−0.4 to 0.0)	0.095
No lipid-lowering agents, mmol/l	1063 (39.2^1^) (80.9^4^)	3.1 (3.1 to 3.2)	3.1 (3.0 to 3.1)	190 (62.5^1^) (92.2^4^)	2.8 (2.7 to 2.9)	2.8 (2.7 to 3.0)	0.2 (0.0 to 0.4)	0.016
Current smoking, *n* (%)	2309 (85.1)	430 (18.6)	18.9 (17.2 to 20.5)	291 (95.7)	54 (18.6)	12.8 (7.1 to 18.4)	6.0 (0.0 to 12.2)	0.050
Using antihypertensive agents, *n* (%)	2713 (100)	1806 (66.6)	–	304 (100)	108 (35.5)	–	–	–
Groups of antihypertensive agents, *n* (%)								
ACE/AII blockers	2713 (100)	1435 (52.9)	54.3 (52.4 to 56.3)	304 (100)	85 (28.0)	23.5 (16.7 to 30.3)	30.8 (23.4 to 38.2)	<0.001
Beta blockers^a^	–	790 (29.1)	–	–	24 (7.9)	–	–	–
Calcium antagonists^a^	–	750 (27.6)	–	–	24 (7.9)	–	–	–
Thiazides^a^	–	741 (27.3)	–	–	30 (9.9)	–	–	–
Number of antihypertensive agents, *n* (%)								
1	–	492 (18.1)	–	–	44 (14.5)	–	–	–
2	–	552 ( 20.4)	–	–	34 (11.2)	–	–	–
≥3	–	762 ( 28.1)	–	–	30 (9.9)	–	–	–
Lipid-lowering medication, *n* (%)	2713 (100)	1399 (51.6)	53.1 ( 51.1 to 55.1)	304 (100)	98 (32.2)	29.0 (22.1 to 35.9)	24.1 (16.6 to 31.5)	<0.001
Lipid-lowering medication with CHD	–	471 (72.9)	75.0 (71.6 to 78.5)	–	26 (89.7)	77.2 (58.3 to 96.1)	−2.2 (−21.6 to 17.3)	0.827
Lipid-lowering medication with no CHD	–	915 (44.9)	46.7 (44.4 to 49.0)	–	70 (27.5)	22.1 (14.6 to 29.5)	24.6 (16.5 to 32.8)	<0.001
Acetylsalicylic acid, *n* (%)	2713 (100)	917 (33.8)	33.2 (31.4 to 35.0)	304 (100)	51 (16.8)	23.6 (17.3 to 29.9)	9.6 (2.8 to 16.4)	0.006

1: % of total population (type 1 or type 2 diabetes).

2: % of subpopulation with or without known CHD.

3: % of subpopulation prescribed lipid-lowering medication.

4: % of subpopulation not prescribed lipid-lowering medication.

a Data only from general practice.

b Average predictions adjusted for age, sex and diabetes duration.

c Average marginal effects.

HbA1c: haemoglobin A1c; CI: confidence interval; LDL: low-density lipoprotein; CHD: coronary heart disease.

Among type 2 diabetes patients, 64.5% were prescribed one or more antihyperglycaemic agents, whereas 35.5% were treated with lifestyle alone. Oral antihyperglycaemic treatment was prescribed to 42.1%. Insulin was used as the only treatment in 12.3% and insulin in combination with other glucose-lowering drugs was used by 10.1%. Furthermore, 20.4% of type 2 diabetes patients were prescribed two antihyperglycaemic agents and 28.1% were prescribed three or more.

### Attained treatment targets

**Figure 2.**
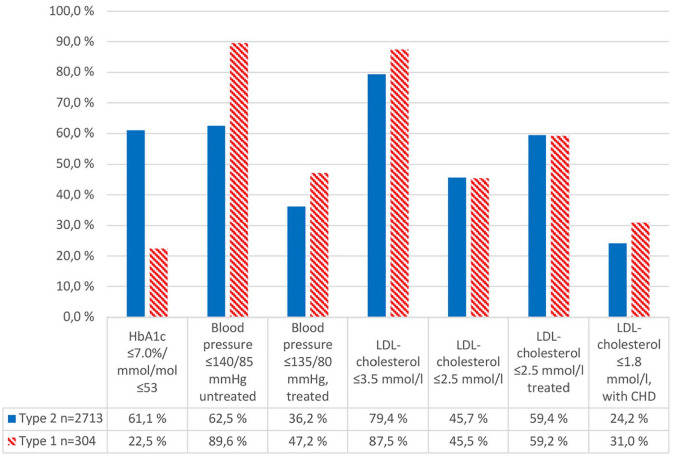
Attained crude treatment targets in all adults with diabetes in Salten, Norway.

Substantially more type 2 diabetes patients than type 1 diabetes patients reached the HbA1c treatment target of 7.0% or less/53 mmol/mol or less, 61.1% versus 22.5% (Figure 2, crude analyses). After adjustments for age, sex and diabetes duration, the difference between diabetes types was reduced to 57.4% versus 45.2% (*P*=0.003).

In patients using antihypertensive agents, 36.2% type 2 and 47.2% type 1 diabetes patients had blood pressure of 135/80 mmHg or less. After adjustments we found no difference between diabetes types (*P*=0.144). If not on medication, type 2 and type 1 diabetes patients differed in the proportion having blood pressure of 140/85 mmHg or less (62.5% vs. 89.6%), and these differences persisted after adjustments (*P*<0.001). In patients using lipid-lowering agents, the treatment target for LDL-cholesterol (⩽2.5 mmol/L) was reached in 59.4% of type 2 and 59.2% of type 1 diabetes patients.

## Discussion

By including all patients with diagnosed diabetes in a geographical area, the present study identifies the true prevalence of diagnosed diabetes in all age groups and diabetes-related vascular complications in adults with type 1 and type 2 diabetes. The total prevalence of type 2 and type 1 diabetes in all age groups was 3.4% and 0.45%, respectively. In adults aged 20 years and older the prevalence of type 2 and type 1 diabetes was 4.4% and 0.49%. CHD was more prevalent in type 2 than in type 1 diabetes, also after adjusting for known confounders, 23.1% versus 15.8%, respectively. Type 2 diabetes patients had higher blood pressure and lower HbA1c than type 1 diabetes patients before and after adjustments. Substantially more type 2 than type 1 diabetes patients reached the HbA1c treatment target even after adjustments, 57.4% versus 45.2%, respectively.

### Prevalence of diabetes

First, our estimates of diabetes prevalence in Salten are lower than global estimates and estimates from the USA and most parts of Europe. A study on US adults (aged ⩾20 years) found a prevalence of type 1 and type 2 diabetes of 0.5% and 8.5%, respectively [11]. A Swedish registry-based study reported a total diabetes prevalence of 4.7% in all age groups in 2012, but had no information on diabetes subtypes [12].

A Norwegian study from 2006 based on self-reported data, had an attendance rate of 56% and reported a prevalence of known diabetes of 4.3% in the age group 20 years and older; 4.9% in men and 3.9% in women [13]. Another study on self-reported diabetes from 2004 reported a prevalence of 2.3% in all age groups, and 3.4% among those aged 30 years and older [14].

We consider a recent Norwegian registry-based study with an estimated prevalence of type 2 diabetes of 6.1% in the age group 30–89 years [3] to be the most relevant comparison for our findings of a slightly lower prevalence of 5.3% in the same age group, although with a slightly higher prevalence of 5.4% based on sensitivity analyses. The registry study was based on national databases only and lacked validation of diagnosis from clinical records. In contrast, in the present study we used information from electronic records in primary and specialist care, with manually validated diagnoses. Our prevalence estimates may therefore be more accurate.

Explanations for the discrepant prevalence findings could, however, also be related to differences in rates of opportunistic screening, undiagnosed cases, trends for underlying risk factors, such as BMI, all-cause mortality in the diabetes population and the ethnic composition when the studies were performed. Many people with type 2 diabetes are still undiagnosed [15].

### Cardiovascular risk factors and complications

Our findings regarding cardiovascular risk factors and complications are generally in line with other studies. A recent systematic review on patients with type 2 diabetes reported that CVD affected 32.2% and 21.2% had CHD [16]. A Swedish study including type 2 diabetes patients requiring glucose-lowering drugs reported a CVD prevalence of 34% [17]. Other studies on type 2 diabetes patients have reported a CVD prevalence of 17% to 23%, 18% in men and 14% in women [7], [18–21]. The differences in crude rates of CVD and CHD between diabetes types can partly be explained by differences in age, sex and diabetes duration, as seen in our adjusted analyses where these differences were less pronounced. Furthermore, the pathophysiological process leading to CVD in type 1 and type 2 diabetes differs [22].

### Attained treatment targets

Only 22.5% of type 1 diabetes patients reached the HbA1c treatment target versus 61.1% of type 2 patients. This is comparable to other studies including both type 1 and type 2 diabetes patients reporting proportions of 52% to 57%, and 54.2% in type 2 diabetes patients [23–26]. We also identified inadequate lipid control as the treatment target for LDL-cholesterol was reached in only 60% in patients receiving lipid-lowering medication.

### Strengths and limitations

The strengths of the present study include the large sample size obtained within an integrated and defined health system and the use of real-world data obtained from both primary and specialist care in a geographically defined area. The diabetes diagnoses were based on the physicians’ clinical diagnoses and validated during data collection. No financial incentives related to pay-for-performance were operating at the time of the study. A limitation may be that we only included patients in primary care who had been in contact with their GP in the period 1 January 2012 to 31 December 2014. This may have excluded some individuals infrequently visiting their GPs.

The prevalence of type 2 diabetes varies considerably between ethnic groups [27,28]. By standardising to the Norwegian immigrant population, the prevalence estimate in Salten only changed by 0.1%. Due to the low number of persons with type 1 diabetes the power to detect differences between diabetes types was limited. Finally, we lack information about individualised treatment targets based on age, multimorbidity and individual preferences.

Identifying gaps in treatment and prevention followed by quality improvement strategies to improve risk factor control may contribute to a further reduction in the individual risk of diabetes complications.

## Conclusion

The present study provides benchmark estimates on the prevalence of diagnosed type 1 and type 2 diabetes in a Norwegian geographically defined population showing a slightly lower prevalence of type 2 diabetes than a recent estimate based on registry data. Glycaemic control was not satisfactory in the majority of patients with type 1 diabetes. CHD and hypertension were more prevalent in patients with type 2 diabetes. Continued monitoring of both diabetes prevalence and diabetes-related risk factors and complications is necessary to target interventions in subgroups in need of more intensive treatment.

## Supplemental Material

SJP951004_Supplementary_Figures – Supplemental material for The total prevalence of diagnosed diabetes and the quality of diabetes care for the adult population in Salten, Norway Click here for additional data file. Supplemental material, SJP951004_Supplementary_Figures for The total prevalence of diagnosed diabetes and the quality of diabetes care for the adult population in Salten, Norway by Kristina B. Slåtsve, Tor Claudi, Knut Tore Lappegård, Anne K. Jenum, Marthe Larsen, John G. Cooper, Sverre Sandberg and Tore Julsrud Berg in Scandinavian Journal of Public Health

SJP951004_Supplementary_Table_1 – Supplemental material for The total prevalence of diagnosed diabetes and the quality of diabetes care for the adult population in Salten, Norway Click here for additional data file. Supplemental material, SJP951004_Supplementary_Table_1 for The total prevalence of diagnosed diabetes and the quality of diabetes care for the adult population in Salten, Norway by Kristina B. Slåtsve, Tor Claudi, Knut Tore Lappegård, Anne K. Jenum, Marthe Larsen, John G. Cooper, Sverre Sandberg and Tore Julsrud Berg in Scandinavian Journal of Public Health
