# Supplementary material for: The total prevalence of diagnosed diabetes and the quality of diabetes care for the adult population in Salten, Norway
Source: Scand J Public Health. 2020 Aug 27;50(2):161–71. doi: 10.1177/1403494820951004 (PMC8873303; doi:10.1177/1403494820951004)

Supplementary Figure 1: Flowchart of patients

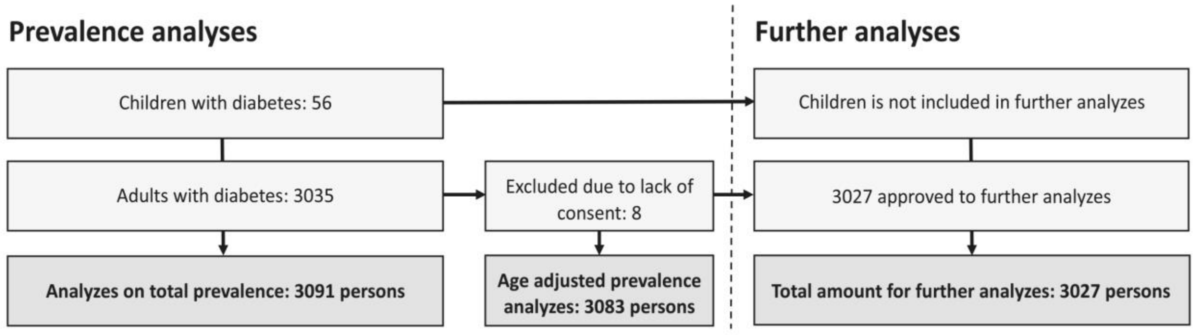

Supplementary Figure 2: Adults patients included in the study: Diabetes type and source of data collection. Shared care includes patients visiting both general practice and specialist care.

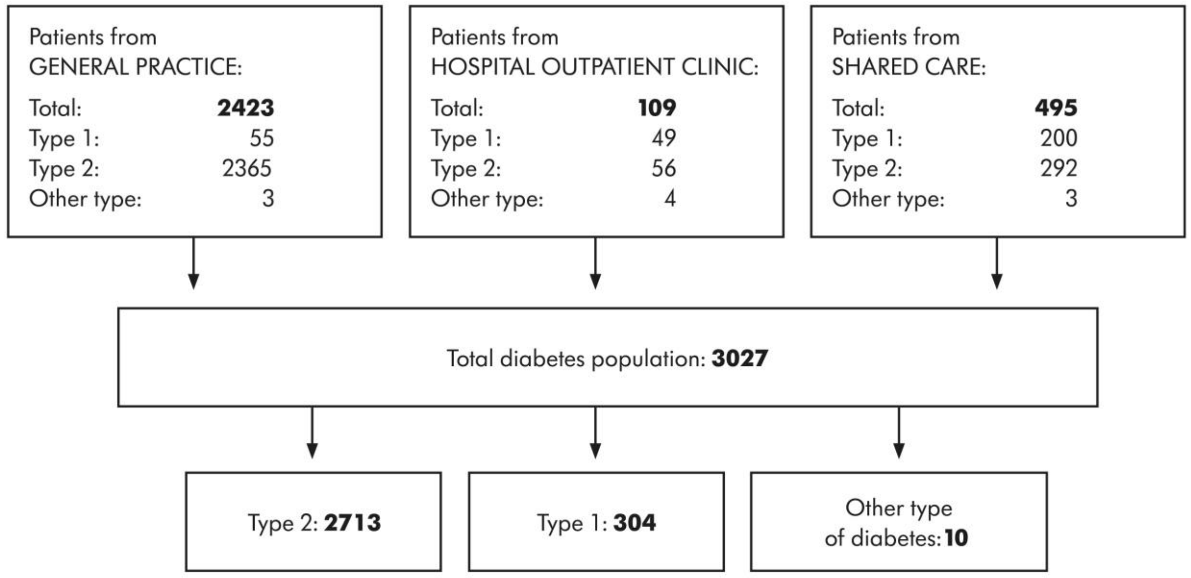

Supplement: SJP951004_Supplementary_Figures – Supplemental material for The total prevalence of diagnosed diabetes and the quality of diabetes care for the adult population in Salten, Norway [file SJP951004_Supplementary_Figures.pdf]
