# Supplementary material for: The total prevalence of diagnosed diabetes and the quality of diabetes care for the adult population in Salten, Norway
Source: Scand J Public Health. 2020 Aug 27;50(2):161–71. doi: 10.1177/1403494820951004 (PMC8873303; doi:10.1177/1403494820951004)
Supplement: SJP951004_Supplementary_Table_1 – Supplemental material for The total prevalence of diagnosed diabetes and the quality of diabetes care for the adult population in Salten, Norway [file SJP951004_Supplementary_Table_1.pdf]

Caption Supplementary Table 1: Variable extraction in general practice and hospital outpatient clinic.

**Variables**	**General practice:**<br>All adults (≥18 years) with a diagnosis of diabetes Jan. 1st, 2012 to Dec. 31st, 2014	**Hospital outpatient clinic**:<br>All adults (≥18 years) visiting Oct. 1st, 2013 to Dec. 31st, 2014
**Characteristics**		
Diabetes duration	2014 minus year of diagnosis	2014 minus year of diagnosis
Height	If ever registered	If ever registered
Weight	15 months	15 months
BMI	15 months	15 months
Current smokers	No; if ever registered as non- smoker.<br>Yes; if registered as current smoker the last 5 years and not changed smoking status	No; if ever registered as non- smoker.<br>Yes; if registered as current smoker the last 5 years and not changed smoking status
**Complications**		
Microvascular complications		
Retinopathy	If ever registered	If ever registered
Reduced foot sensibility	If ever registered	15 months
Macrovascular complications		
Coronary heart disease	If ever registered	If ever registered
Stroke	If ever registered	If ever registered
Diabetic foot ulcer	If ever registered	If ever registered
**Processes of care**		
HbA1c	15 months	15 months
Blood pressure	15 months	15 months
Lipids	36 months	36 months
Creatinine/eGFR	36 months	36 months
Microvascular screening		
Reduced foot sensibility	15 months	15 months
Eye examination	24 months	24 months
Medication	Prescriptions 15 months and if registered manually	If registered manually

Retinopathy: Non-proliferative and proliferative retinopathy.
Reduced foot sensibility: Pathological monofilament test and/or any form of vibration test.
Coronary heart disease: Acute myocardial infarction, angina, percutaneous coronary intervention/coronary artery bypass surgery.

Stroke: Excluding transient ischemic attacks.
15 months: Oct. 1st, 2013 to Dec. 31st, 2014.
24 months: Jan. 1st, 2013 to Dec. 31st, 2014.
36 months: Jan 1st, 2012 to Dec. 31st, 2014.
